# Aortic Aneurysm Eroding into the Spine

**DOI:** 10.1055/s-0038-1669416

**Published:** 2019-01-25

**Authors:** T. Konrad Rajab, Miriam W. Beyene, Farhang Yazdchi, Matthew T. Menard

**Affiliations:** 1 Division of Vascular and Endovascular Surgery, Department of Surgery, Brigham and Women's Hospital and Harvard Medical School, Boston, Massachusetts; 2 Brandeis University, Waltham, Massachusetts

**Keywords:** aortic aneurysm, rupture, contained rupture, surgery

## Abstract

Aortic aneurysms are usually asymptomatic until catastrophic rupture occurs. Ruptured abdominal aortic aneurysms classically present with acute back pain, shock, and a pulsatile abdominal mass. The natural history of some aortic aneurysms also includes a stage of contained rupture. This occurs when extravasation of blood from the ruptured aneurysm is contained by surrounding tissues. Here, the authors report the case of a chronic contained abdominal aortic aneurysm rupture that resulted in erosion of the spine.

Abdominal aortic aneurysms are usually asymptomatic until catastrophic rupture occurs. 1 Ruptured abdominal aortic aneurysms classically present with acute back pain, shock, and a pulsatile abdominal mass. 2 The natural history of some aortic aneurysms also includes a stage of contained rupture. This occurs when extravasation of blood from the ruptured aneurysm is contained by surrounding tissues. Here, we report the case of a chronic contained abdominal aortic aneurysm rupture that resulted in erosion of the spine.

A 76-year-old male smoker presented with progressive back pain and a pulsatile abdominal mass. He had a history of coronary artery disease, hypertension, hyperlipidemia, and chronic obstructive pulmonary disease. Physical examination revealed an expansile abdominal mass and diminished femoral pulses. Laboratory testing was notable for a normal white blood cell count, erythrocyte sedimentation rate, and creatinine. Computed tomography demonstrated a 14 cm juxtarenal abdominal aortic aneurysm ( Fig. 1 ) eroding into the third and fourth lumbar vertebrae ( Figs. 1 and 2 ). There was also severe iliofemoral occlusive disease. The patient was taken to the operating room for urgent open aortic aneurysm repair. Incision of the aneurysm sac revealed eroded vertebral bodies that were exposed within the aortic lumen ( Fig. 3 ). There was moderate atherosclerosis but no obvious inflammation. Microbiology specimen did not grow any organisms. The aneurysm was repaired with an aortobifemoral reconstruction using a bifurcated Dacron graft. The postoperative course was unremarkable and the patient was discharged to rehabilitation on postoperative day 5. Orthopaedic consultation recommended conservative management of the spinal erosion without a need for operative stabilization. He was at his baseline 12 months following his repair.

**Fig. 1:**
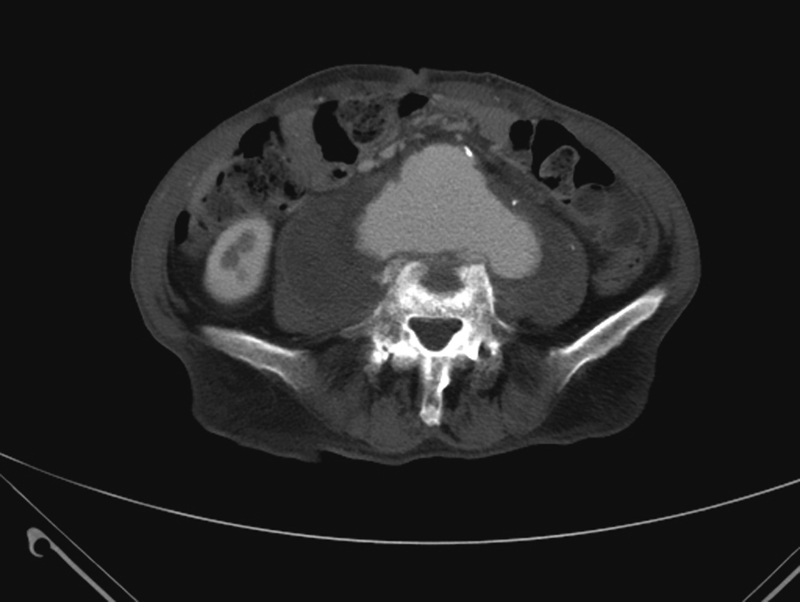
Computed axial tomography shows the 14 cm abdominal aortic aneurysm with mural thrombus.

**Fig. 2:**
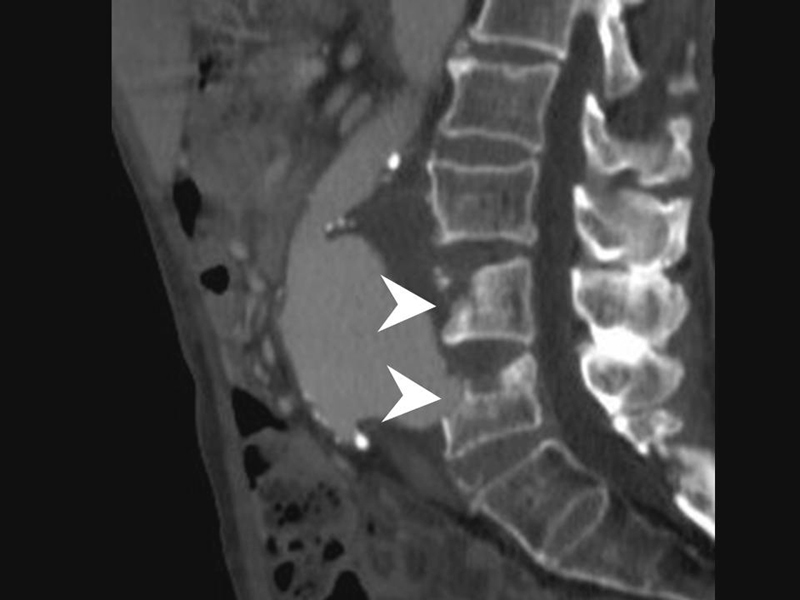
Bone window images of the sagittal reconstruction show the aneurysm eroding the spine (arrowheads).

**Fig. 3:**
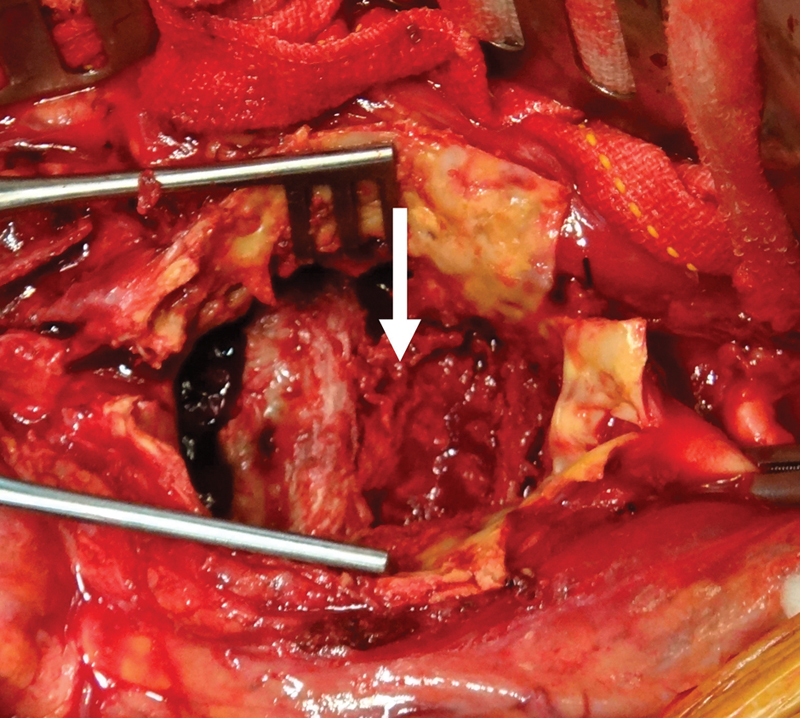
Intraoperative photograph shows the incised aneurysm sac (arrow), and eroded vertebral bodies that are exposed inside the aortic aneurysm.

Ruptured aortic aneurysm has a high mortality rate. In a large prospective series of patients with ruptured aneurysms, 25% of patients died before reaching a hospital and 51% died at the hospital before undergoing surgery. 3 The consequences of abdominal aortic aneurysm rupture depend on the anatomic location of the rupture. Free rupture into the peritoneal cavity is rapidly fatal. Contained rupture occurs when extravasation of blood is contained by surrounding tissues. Depending on the quality of the surrounding tissue, chronic contained rupture may be present over a prolonged period of time. The duration of this stage depends on the strength of the tissue containing the hematoma. Posterior extension of the rupture can erode the spine, as occurred in this patient and others described in the literature. 4 5 Alternatively, local extension of vertebral osteomyelitis or discitis can cause mycotic aortic aneurysm formation. These pathologies result in atypical presentations of abdominal aortic aneurysms.
